# Clinical characteristics of adult inpatients with Measles in Beijing from 2010 to 2021: a retrospective analysis

**DOI:** 10.1186/s12879-023-08256-2

**Published:** 2023-05-09

**Authors:** Lixue Zhao, Yu Wang, Xue Chen, Liu Yang, Miaotian Cai, Zhili Zhang, Yulin Zhang, Yingmin Ma

**Affiliations:** 1 Department of Respiratory and Critical Care Medicine, Beijing Youan Hospital, Capital Medical University, Beijing, 100069 China; 2 Department of Respiratory and Critical Care Medicine, Beijing LiangXiang Hospital, Capital Medical University, Beijing, 102401 China

**Keywords:** Measles, Clinical characteristics, Infectious diseases, epidemiology, Retrospective analysis

## Abstract

**Background:**

With the measles vaccine coverage rate gradually increasing, adult patients’ epidemiological and clinical characteristics have changed.

**Aims:**

To analyze the clinical characteristics of adult measles patients in Beijing Youan Hospital.

**Methods:**

We retrospectively reviewed the electronic medical records of 818 patients diagnosed with measles at Beijing Youan Hospital between June 2010 and October 2021. We divided all hospitalized patients into two demographics groups, using 14 years of age as the cut-off.

**Results:**

Of the adult inpatients, 110 (74.83%) were aged 20–40. There was an overall peak incidence in 2014, and yearly peaks came in April. Fever, cough, erythema, and Koplik’s spots were present in 79.59%, 82.1%, 99.3%, and 59.8% of the adult group, respectively, compared to 75.26%, 92.0%, 99.9%, and 39.0% of the pediatric group. Decreased lymphocytes and hepatic impairment were common in adults. The adult group’s median level of C-reactive protein was higher than that of the pediatric group (p < 0.05). The positive rate of measles antibody (IgM) detection was 64.6% in the adults and 78.8% in the pediatric group (p < 0.05). Of the adults, 46.9%, 8.8%, and 66% had pneumonia, gastroenteritis, and antibiotic use, compared to 89.6%, 2.7%, and 83.2% of the pediatric patients. The duration of symptoms before admission and the average length of hospital stay was approximately six days in both groups.

**Conclusions:**

Koplik’s spots are more likely to be detected by clinicians in adult patients admitted to the hospital. Active surveillance is helpful for adults who are negative for IgM on admission. Although the proportion of adult measles patients with liver injury is high, the disease is generally mild. Measles significantly impacts peripheral blood lymphocytes in adults, but adults are at lower risk of concurrent pneumonia than the pediatric group. Clinicians need to pay attention to the appropriate use of antibiotics. Expanding the coverage of the measles vaccination in high-risk areas is beneficial for preventing measles in adults.

## Introduction

The measles disease, caused by the measles virus, is one of the most common acute respiratory infectious diseases. It leads to epidemics in densely populated and unvaccinated areas, with an average of one pandemic every 2–3 years [1]. There is no specific antiviral treatment for measles, and the most effective measure to prevent the disease is vaccination against the measles virus [2]. In China, measles occurs throughout the year, and its incidence peaks between March and May. Children aged between 6 months to 5 years are most susceptible to measles virus infection [3]. In recent years, the proportion of measles cases in infants younger than 8 years has increased considerably. Measles cases in children over 14 years of age accounted for 26.9% of all cases in 2013, which increased to 41.6% in 2019 [4]. Most complications in infected patients occurred in children younger than 2 years, with the common complications observed in both children and adults being pneumonia and diarrhea [5]. Hepatitis was common only in adults aged older than 20 years [6]. To date, detailed data analyzing the epidemiology, complications, and biochemical parameters of adult patients with measles infection are limited. In this study, we summarized the clinical data of 818 hospitalized patients with measles in Beijing Youan Hospital over the past 12 years, to investigate their clinical characteristics.

## Materials and methods

### Data collection

This retrospective study was conducted using the electronic medical records of 818 hospitalized patients diagnosed with measles who were admitted to Beijing Youan Hospital between June 2010 and October 2021. All patients met the “Diagnostic criteria of measles” (WS296-2017), a health industry standard of the People’s Republic of China [7]. Patients included were either clinically or laboratory-confirmed cases. According to the age range of pediatric diagnosis and treatment in China, we divided all hospitalized patients into two groups using 14 years of age as the cut-off age standard, with those aged 14 years and younger comprising the pediatric group and those aged more than 14 years consisting of the adult group. The clinical presentations of the included patients were documented on admission, and any complications were noted in patient information at discharge. We performed all laboratorial and imageologic examinations on patient admission. Routine peripheral blood tests referred to “Reference intervals of blood cell analysis for children” (WS/T 779–2021), a health industry standard of the People’s Republic of China [8]. Clinical biochemistry tests referred to “Reference intervals of clinical biochemistry tests commonly used for children” (WS/T 780–2021), the health industry standard of the People’s Republic of China [9]. Our study used enzyme-linked immunosorbent assay (ELISA), a qualitative test, to detect measles-specific antibodies (IgM).

### Ethical approval

The study was approved by the Ethics Committee of Beijing Youan Hospital Capital Medical University. All experiments were performed in accordance with relevant guidelines and regulations (e.g., Declaration of Helsinki), and informed consent was obtained from all subjects and/or their legal guardian(s).

### Statistical analysis

SPSS 25.0 and GraphPad Prism 8.0 were used for statistical analyses. Measurement data were expressed as mean or median, and Student’s t-tests or Mann–Whitney U tests were used for comparison. Enumeration data were expressed as rates, and the Chi-squared test was used for analysis. A two-sided p-value of < 0.05 was considered statistically significant.

## Results

### Epidemiological characteristics

**Fig. 1:**
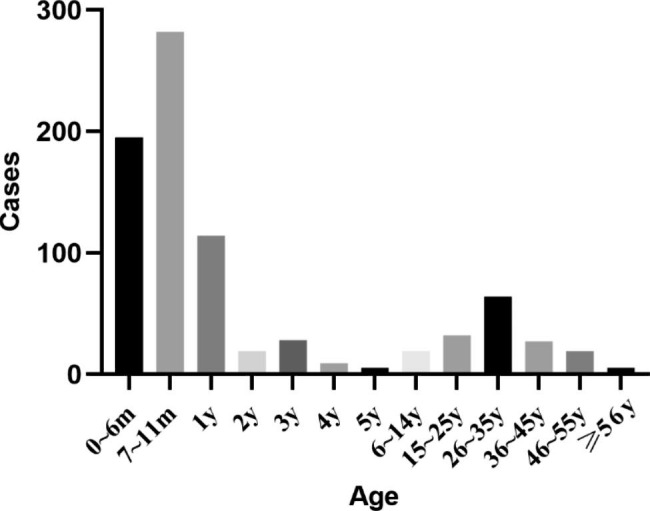
The age distribution of inpatients with measles in Beijing Youan Hospital between 2010–2021

Age was categorized into two groups: (1) 671 children, median age of 8 months (range: 1 month-14 years), 464 males; (2) 147 adults, median age of 30 years (range: 15–61 years), 72 males. The pediatric group accounted for 82.03% of the total inpatients, of which 69% were males, while there was less of a sex difference in the adult group. Children younger than two years of age accounted for 72.25% of the total patients (Fig. 1). While adult patients accounted for 17.97% of investigated patients, age at onset was 20–40 years in most cases, accounting for 74.83% of the adult patient group. Community morbidity was 96.72% in the pediatric group and 91.84% in the adult group (p < 0.05; Table 1).

**Table 1:** The age distribution and basic information of patients with measles

	Pediatric (≤14y) n = 671				Adult (>14y) n = 147						*P*-value
	0~6m	7~12m	1~5y	6~14y	15~25y	26~35y	36~45y	46~55y		>56y	
**Group n(%)** ^**1**^	195 (23.84)	282 (34.47)	175 (21.39)	19 (2.32)	32 (3.91)	64 (7.82)	27 (3.30)		19 (2.32)	5 (0.61)	--
**Age [*M*(*P*25**, ***P*75)]**	8m (6, 12m)				30y (26, 39y)						--
**Male (n, %)** ^**2**^	464 (69.15)				72 (48.98)						≤0.05
**Community-onset (n, %)**	649 (96.72)				135(91.84)						≤0.05
**Comorbidities (n, %)** ^**2**^	69 (10.28)				25 (17.01)						≤0.05
**Congenital heart disease** ^**3**^	33 (4.92)				3 (2.04)						0.123
**Diabetes**	1 (0.15)				6 (4.08)						≤0.05
**Autoimmune disease** ^**4**^	5 (0.75)				6 (4.08)						≤0.05
**Gastrointestinal disease** ^**5**^	9 (1.34)				0 (0.00)						0.329
**Neurological diseases** ^**6**^	9 (1.34)				2 (1.36)						1.000
**Hepatitis B**	4 (0.60)				4 (2.72)						0.056
**Tumour** ^**7**^	3 (0.45)				2 (1.36)						0.482
**Others** ^**8**^	5 (0.75)				2 (1.36)						0.811

Abbreviations:

^1^n (each age group) / N (all patients)

^2^Incidence in the pediatric or adult patients with measles

^3^ Congenital heart disease: Congenital atrial septal defect, Congenital ventricular septal defect, Patent foramen ovale

^4^ Autoimmune disease: Kawasaki disease,Thyroid dysfunction, Nephrotic syndrome, Thrombocytopenic purpura

^5^ Gastrointestinal disease: Congenital hirschsprung’s disease, Biliary atresia, Anal atresia

^6^ Neurological diseases: Hydrocephalus, Cerebral infarction, Cerebral hemorrhage, Epilepsy, Motor neuron damage, Myelin sheath development retardation

^7^ Tumour: Rectal cancer, Ovarian cancer, Juventus sarcoma, Retroperitoneal neuroblastoma

^8^ Others: Asthma, Pulmonary dysplasia, Aplastic anemia, Tuberculosis, AIDS.

**Fig. 2:**
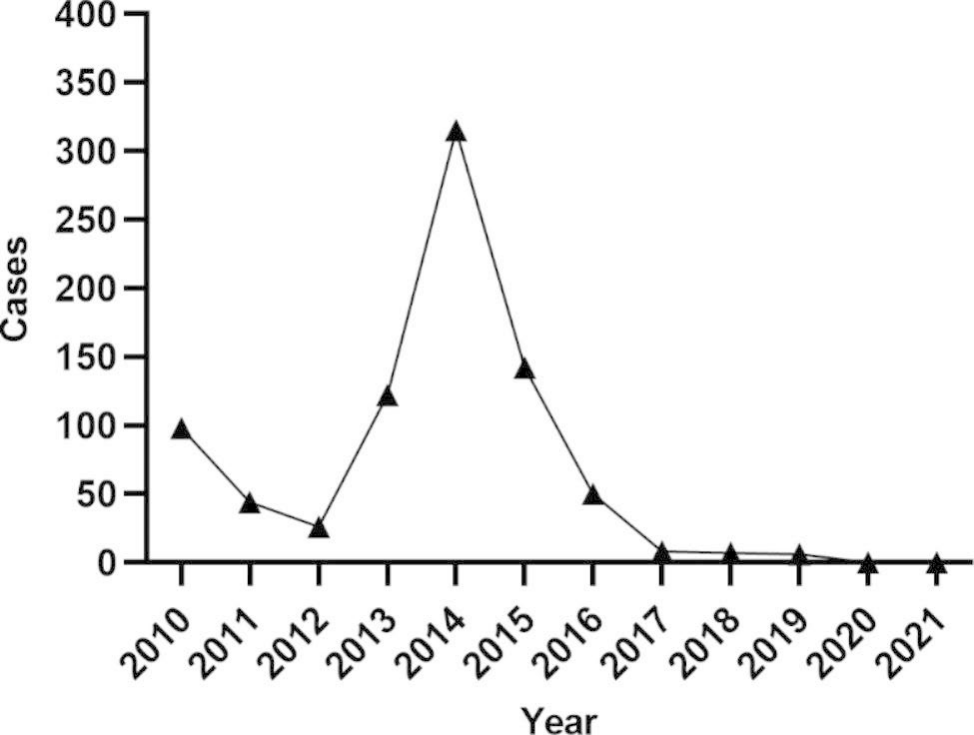
Statistical trends in inpatients with measles in Beijing Youan Hospital between 2010–2021. There was a peak in the incidence of measles in 2014

**Fig. 3:**
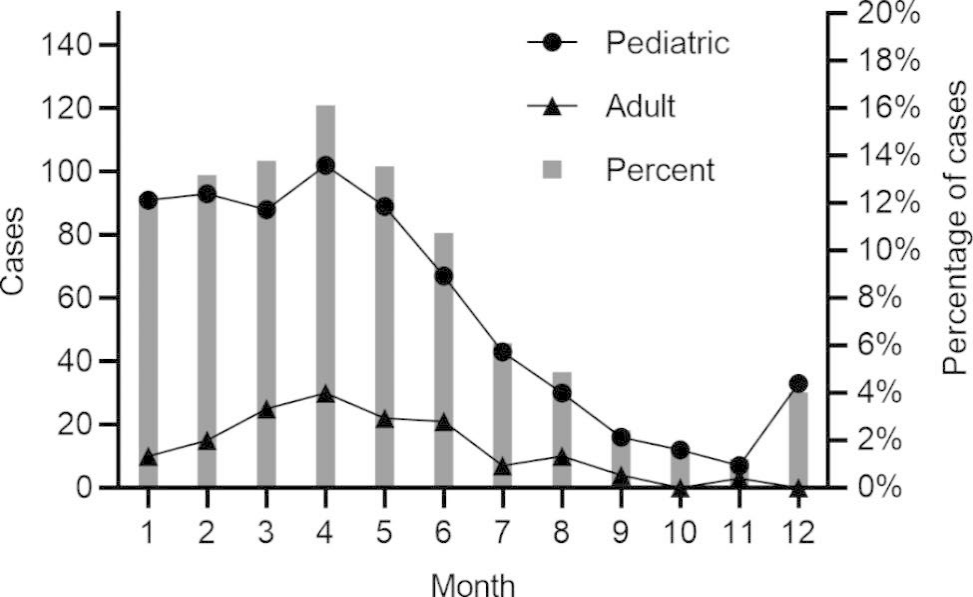
Characteristics of the seasonal distribution of adult and paediatric patients with measles in Beijing Youan Hospital between 2010–2021. The peak of hospitalization in both groups was in April

The number of patients with measles in our hospital decreased between 2010 and 2012. However, in 2013, there was a surge in the measles incidence rate; the number of patients with measles increased to 308, reaching a peak in 2014. There were five hospitalized patients with measles in 2019, and no patients were identified between 2020 and 2021 (Fig. 2). Generally, the number of infected patients increased in December and decreased after April of the following year. The pediatric group experienced a rapid increase in measles cases starting in December each year. The surge in the adult group began slightly later, with cases beginning to increase in January. Nevertheless, the peak of hospitalization in both groups was in April, with significant seasonality (Fig. 3).

### Clinical characteristics and complications

In our study, 10.28% of the pediatric patients had underlying diseases, including congenital heart disease, which accounted for 47.83%. In the adult inpatient group, 17.01% had underlying diseases, including autoimmune diseases and diabetes accounting for 48% (Table 1).

Fever, cough, and systemic rash were the main clinical symptoms in the two groups. Conjunctivitis and Koplik’s spots were common, and the incidence of Koplik’s spots in the adult group was significantly higher than in the pediatric group (p < 0.05). Complications of pneumonia were common in both groups, with the incidence being significantly higher in the pediatric group (p < 0.05). Pneumonia caused by viral infections was generally more common than pneumonia from bacterial infections in hospitalized patients. The risk of concurrent gastroenteritis was higher in the adult group than in the pediatric group (p < 0.05). It was generally rare to find patients with respiratory failure, myocarditis, or measles encephalitis (Table 2).

**Table 2:** Clinical presentation, complications, management, and outcomes in pediatric and adult groups

	Pediatric (≤14y) (N=671)	Adult (>14y) (N=147)	P-value
**Clinical presentation (%)**			
Fever^1^	75.26	79.59	0.265
Cough	92.00	89.10	0.265
Rhinorrhea	15.10	10.90	0.191
Rhinobyon	0.30	0.70	1.000
Conjunctivitis	28.00	27.90	0.975
Erythra	99.90	99.30	0.795
Koplik’s spots	39.00	59.80	≤0.05
Stimson	0.40	0.70	1.000
**Complications (%)**			
Gastroenteritis	2.70	8.80	≤0.05
Measles encephalitis	2.40	0.70	0.321
Pneumonia	89.60	46.90	≤0.05
Pneumonia (bacterial)	21.61	7.48	≤0.05
Pneumonia (viral)	67.96	39.46	≤0.05
Myocarditis	2.40	1.40	0.648
**Management and outcomes**			
Death (%)	0.60	0.00	0.775
Antibiotic treatment (%)	83.20	66.00	≤0.05
Antiviral treatment (%) Days^2^ median (IQR)	13.70 6.00 (5,7)	13.70 6.00 (5,7)	0.997 0.740
Days^3^ median (IQR)	6.00 (5,8)	6.00 (4,7)	≤0.05

^1^ Fever on admission

^2^ Duration of symptoms before admission

^3^ Length of hospital stay

### Laboratory Findings

The proportion of patients with elevated white blood cell count and thrombocytopenia was low in both groups. The proportion of decreased lymphocyte counts was higher in the adult group than in the pediatric group. The c-reactive protein level was higher in the adult group than the pediatric group (p < 0.05), suggesting that adults with measles have a more severe inflammatory response. Liver function tests (alanine transaminase) revealed that liver injury was common in adult patients. Renal dysfunction occurred in a low proportion of patients in both groups. The pediatric group had a higher positive rate of measles antibody detection (IgM) (Table 3).

**Table 3:** Auxiliary examinations in pediatric and adult groups

[*M*(*P*25, *P*75)] OR (%)	Pediatric (≤ 14y)	Adult (>14y)	*P*-value
**WBC (×10** ^**9**^ **/l)**	7.5 (5.3, 10.5)	4.9 (3.7, 7.5)	≤0.05
**NE (×10** ^**9**^ **/l)**	3.4 (2.0, 5.2)	4.0 (2.5,6.2)	≤0.05
**LY** **(×10** ^**9**^ **/l)**	3.0 (1.9, 4.5)	0.7 (0.5, 1.0)	≤0.05
**PLT (×10** ^**9**^ **/l)**	280.0 (210.1, 363.2)	158.3 (128.2, 203.4)	≤0.05
**CRP** ^**a**^ **(mg/l)**	7.0 (3.0, 18.0)	21.0 (14.0, 47.5)	≤0.05
**TBLB (umol/l)** **DBLB (umol/l)**	5.4 (4.2, 6.8) 1.3 (0.8, 1.9)	12.0 (8.8, 16.0) 3.0 (1.8, 5.5)	≤0.05 ≤0.05
**ALT (u/l)** **AST (u/l)** **TP (g/l)** **ALB (g/l)** **CK (u/l)**	26.0 (19.0, 39.0) 58.0 (48.0, 73.0) 61.8 (58.4, 65.6) 39.6 (36.8, 41.8) 66.0 (48.0, 100.0)	54.0 (32.0, 146.0) 58.0 (39.0, 107.0) 68.2 (63.8, 72.9) 39.5 (35.4, 42.7) 154.5 (80.0, 292.0)	≤0.05 0.956 ≤0.05 0.665 ≤0.05
**CKMB** **(ng/ml)** **BUN (mmol/l)**	3.2 (1.4, 20.0) 2.2 (1.5, 1.9)	1.7 (0.5, 11.5) 3.5 (2.7, 4.5)	≤0.05 ≤0.05
**SCr** **(umol/l)**	27.0 (22.0, 32.0)	67.0 (56.0, 84.0)	≤0.05
**IgM** ^**b**^ **(%)**	78.8	64.6	≤0.05

Abbreviations: WBC: White Blood Cell; NE: Neutrophil; LY: Lymphocyte; PLT: Platelet; CRP: C-Reactive Protein; TBLB: Total Bilirubin; DBLB: Direct Bilirubin; ALT: Alanine Aminotransferase; AST: Aspartate transaminase; TP: Total Serum Protein; ALB:Serum Albumin; CK: Creatine Kinase; CKMB: Creatine Kinase Isoenzymes; BUN: Blood Urea Nitrogen; SCr: Serum Creatinine

^a^Normal range 0−10 mg/l

^b^Positive Rate of Measles Virus-Specific IgM Antibodies

### Management and outcomes

The overall prognosis for the two groups was good, and the probability of severe disease or even death was low. Four patients who died during the study included children aged approximately 1 year, two patients with congenital heart disease/heart failure, one with Behçet’s disease, and one with severe pneumonia. Notably, all of the four patients who died exhibited pneumonia. The proportion of antibiotic use was high in both groups, and was significantly higher in the pediatric group (p < 0.05). Bacterial infection was identified in 29.66% of the pediatric group, compared to 10.20% of the adult group. The median length of hospital stay was approximately six days in both groups (Table 2).

## Discussion

Measles continues to be a highly contagious disease that threatens human health. With the gradual increase in the coverage rate of the two-dose measles vaccine, the epidemiological and clinical characteristics of measles patients have changed. In this study, we described the demographic and clinical features of patients diagnosed with measles at the Beijing Youan Hospital over the past 12 years, and identified their age-group-related characteristics. As an infectious disease hospital, Beijing Youan Hospital admits patients from all over the country, therefore, the case data are to a certain degree representative of the Chinese population.

Most measles cases occur during late winter and early spring in temperate zones. Patients with measles admitted to our hospital more commonly in winter and spring, with the peak incidence occurring in April. China began mass use of the measles vaccine in 1965, after which incidence levels of measles infection declined. In 1986, China introduced a two-dose schedule using this vaccine at 8 months and 7 years of age, respectively. Eventually, in 2005, the timing of measles-containing vaccine 2 (MCV2) administration was changed from 7 years to 18–24 months of age [10]. In 2005, all children were required to undergo a check of their vaccination records when entering kindergarten and primary school. Our data showed that the number of patients hospitalized for measles was significantly higher in 2014 than in other years. Other studies also estimated the measles incidence in China between 2013 and 2019 and found that it peaked in 2014 [4]. Community morbidity accounted for more than 90% of all admissions, with its incidence in the pediatric group being slightly higher than in the adult group. The medical and economic indicators in Beijing are relatively high, but there are many mobile populations in Beijing, which increases the risk of infection. In our study, children aged ≤ 1 year accounted for 72.25% of the total patients, and most were hospitalized due to complications such as pneumonia. Since the first dose of the measles vaccine is administered at 8 months of age, and the seroconversion rate is about 80–85% [11], we should focus on monitoring children before the second dose of the vaccination in order to achieve timely detection and early isolation. An extra dose of MCV should be administered to 6-month-old infants at a high risk of contracting measles. It should be noted that the age distribution of measles shifts further towards adolescence and adulthood as vaccination coverage and population immunization levels continue to improve [12]. A lower seropositivity rate of measles IgG antibodies has been reported in adults, which increases the risk of measles infection [13]. Relevant policies should allow adolescent populations who missed the MCV to receive the vaccine when necessary [14].

Patients in both groups had a lower probability of having underlying diseases, which indicates that population susceptibility to the measles virus has a minimal association with underlying diseases. Symptoms such as fever, systemic rash, cough, conjunctivitis, and rhinorrhoea were common in the two groups of patients, and there was no significant difference between them. In our study, Koplik’s spots, one of the characteristic symptoms of measles infection, were more common in the adult group. This phenomenon is related to the immune statuses of infected patients. Foreign studies have reported that pathognomonic Koplik’s spots can be found in 70% of patients with measles [15]. Our study suggested a low incidence of Koplik’s spots, which was considered to be associated with a longer duration of onset on admission in both groups. It should be noted that Koplik’s spots may also appear in rubella or other viral infections [16]. The positive rate of measles virus-specific IgM antibodies was lower in the adult group. The early detection of measles virus-specific IgM antibodies may be low [17], but almost all measles show positive test results 4 days after rash eruptions [18]. Active surveillance of measles-specific antibodies is essential in adult patients.

The total lymphocyte count is reduced during the acute phase of measles infection, increasing susceptibility to other infections [19]. Measles infection may also increase mortality due to other infectious diseases for about 2–3 years following infection [20]. Infants and undernourished children have an increased risk of complications due to measles infection [15]. Our data showed that the risk of complicated pneumonia and the use of antibiotics was higher in the pediatric group than in the adult group. Antibiotics have beneficial effects in preventing complications such as pneumonia, suppurative otitis media, and tonsillitis in children with measles [21]. Nevertheless, prophylactic antibiotic therapy for infected patients remains controversial among infectious disease specialists [22]. Antibiotics are generally recommended for pneumonia, sepsis, or other secondary bacterial complications [15]. In our study, bacterial infection was identified in 29.66% of the pediatric group, compared to 10.20% of the adult group. We found that a significant proportion of measles patients used antibiotics without a clear indication. The rational use of antibiotics in measles patients requires the attention of clinicians. Hepatic involvement in such patients is uncommon [23]. Pediatric patients appear to have a lower risk of hepatic dysfunction [24]. Elevations in transaminase levels are common, representing a direct viral cytopathic effect [25]. Most of these conditions appear in the early stages of the disease, and have positive prognoses. Through our analysis and statistical review of peripheral blood routine and liver and kidney function tests, we found that measles has a more significant impact on adult patients than children, possibly because the protective efficacy of the measles vaccine is reduced over time.

There were some limitations to this study. First, we have not researched information on inpatients infected with measles since 2020, due to the changes in hospital policies following the coronavirus disease 2019 pandemic. Second, our data were all obtained from hospitalized infected patients. Third, the data on specific medication and vaccination of patients with measles were not recorded in detail.

## Conclusion

The clinical characteristics of measles in adults have some differences compared to those of pediatric patients, which is essential for informing future prevention, diagnosis, and treatment strategies for measles infection. Koplik’s spots were more prevalent in adult patients than pediatric ones; therefore, clinicians should pay attention to the early detection of this clinical manifestation. Dynamic monitoring of measles-specific antibodies (IgM) is vital in adult patients. Minor liver injury is common in adult measles patients, but the prognosis is typically positive. Measles infection has a more significant impact on peripheral blood lymphocytes in adults. However, the risk of concurrent bacterial pneumonia is not high, and clinicians need to avoid the prophylactic use of antibiotics. Supplementary measles vaccination for high-risk adults is warranted.

## Data Availability

All data generated or analyzed during this study are included in this published article.

## List of Abbreviations

MCV2: Measles-containing vaccine 2
ELISA: Enzyme-linked immunosorbent assay
